# Effect of Arsenic on Fluoride Tolerance in *Microbacterium paraoxydans* Strain IR-1

**DOI:** 10.3390/toxics11110945

**Published:** 2023-11-20

**Authors:** Megha Mathur, Neha Rawat, Tanushree Saxena, Renu Khandelwal, Neha Jain, Mukesh K. Sharma, Medicherla K. Mohan, Pradeep Bhatnagar, Swaran J. S. Flora, Pallavi Kaushik

**Affiliations:** 1 Applied Microbiology Laboratory, Centre for Rural Development and Technology, Indian Institute of Technology, Delhi 110016, India; meghs2610@gmail.com; 2 Department of Life Sciences, IIS University, Mansarovar, Jaipur 302020, Indiapradeep.bhatnagar@iisuniv.ac.in (P.B.); 3 Centre for Advanced Studies, Department of Zoology, University of Rajasthan, Jaipur 302004, India; 4 Department of Zoology, S.P.C., Government College, Ajmer 305001, India; 5 Department of Biotechnology and Bioinformatics, Birla Institute of Scientific Research, C Scheme, Jaipur 302001, India; kmohan@bisr.res.in; 6 National Institute of Pharmaceutical Education and Research-Raebareli, Lucknow 226002, India

**Keywords:** antagonistic effect, *Microbacterium* sp., minimum inhibitory concentration, toxicity unit, arsenic, fluoride

## Abstract

Fluoride (F) and arsenic (As) are two major contaminants of water and soil systems around the globe, causing potential toxicity to humans, plants, animals, and microbes. These contaminated soil systems can be restored by microorganisms that can tolerate toxic stress and provide rapid mineralization of soil, organic matter, and contaminants, using various tolerance mechanisms. Thus, the present study was undertaken with the arsenic hyper-tolerant bacterium *Microbacterium paraoxydans* strain IR-1 to determine its tolerance and toxicity to increasing doses of fluoride, either individually or in combination with arsenic, in terms of growth inhibition using a toxicity unit model. The minimum inhibitory concentration (MIC)and half maximal inhibitory concentration (IC_50_) values for fluoride increased, from 9 g/L to 11 g/L and from 5.91 ± 0.1 g/L to 6.32 ± 0.028 g/L, respectively, in the combination (F + As) group. The statistical comparison of observed and expected additive toxicities, with respect to toxicity unit (TU difference), using Student’s *t*-test, was found to be highly significant (*p* < 0.001). This suggests the antagonistic effect of arsenic on fluoride toxicity to the strain IR-1. The unique stress tolerance of IR-1 ensures its survival as well as preponderance in fluoride and arsenic co-contaminated sites, thus paving the way for its possible application in the natural or artificial remediation of toxicant-exposed degraded soil systems.

## 1. Introduction

Arsenic (As) and fluoride (F) are two major environmental toxicants that pose their toxicity to all exposed living organisms. Their worldwide co-occurrence in groundwater, surface water, soil, and sediments is contributed to by geogenic and anthropogenic sources [1,2]. The geogenic sources include natural weathering and dissolution from rocks and volcanic activities [1,3,4]. Anthropogenic activities, like mining, coal combustion, and the industrial production of fluorinated and arsenic-containing compounds, add to the natural levels of these toxicants [5,6].

The co-occurrence and toxicity threats of As and F have been realized in many regions of the world, such as China [7], India [8], Mexico [9], Latin America [10], Pakistan [11], Mongolia [12], and Korea [13].

In nature, fluoride exists as organic or inorganic fluorine compounds, with high reactivity and electronegativity [14]. After the intake of fluoride through food or water, it rapidly reaches the blood through gastrointestinal absorption [15]. Although most fluoride is excreted through urine, chronic exposure to it results in gradual retention in bones and teeth, causing skeletal and dental fluorosis [16]. It also induces nephrotoxicity, neurotoxicity, and neuro-physiological disturbances [17,18,19].

Arsenic is another co-existing natural toxic metalloid, a well-known poison ubiquitous in the environment. The complex reactivity of arsenic is evident with the presence of various oxidation states (for example, −5, −3, 0, and +3) in organic and inorganic compounds. The trivalent (+3) and inorganic forms are more toxic than the organic arsenic compounds and those in other states of oxidation [20]. Arsenic exposure in humans may cause neurological problems, cardiovascular diseases, hyperkeratosis, hypertension, reproductive toxicity, gangrene, and diabetes mellitus, as well as lung, bladder, and kidney cancer, etc. [20]. Arsenic shows its detrimental effects on protein metabolism by reacting with its sulfhydryl groups, resulting in loss/deviation in protein activity [21]. Co-exposure to these toxicants (As and F) exhibits mild to severe toxicological implications, like impaired neurological development and memory loss [22], nephrotoxicity, and the disordering of serum metabolites and the gut biome [23].

The impact of As and F on microbial communities is also significant, as these are the major role players in ecological systems for shaping microbial communities [24,25]. The contaminant loads cause the microbial community to shift from sensitive to tolerant forms, which have developed specific adaptive mechanisms. The tolerance to As and F in microbes can be achieved by removing, reducing, or restricting the toxicant’s entry into the cell or by chemical modification to reduce its toxicity [26].

Various researchers have studied the chromosomal- or plasmid-borne genes responsible for such tolerance mechanisms. The F-resistant *crcB* gene has been identified and studied in the fluoride-resistant bacteria *Pseudomonas aeruginosa*, and *Enterobacter* sp., which have been reported to function as fluoride transporters [27,28]. Similarly, arsenic resistance has been reported to be regulated by *ars* genes [29],and the biotransformation of arsenic oxidation forms into one another is regulated by the *aio* operon, including regulatory genes like *aioX* (encodes for periplasmic AsIII-binding proteins), *aioS* (encodes for sensor histidine kinases), and *aioR* (encodes transcriptional regulators), as well as the functional genes *aioB* and *aioA*, which encode for the small and large catalytic subunits of the AsIII oxidase enzyme, respectively. Other genes like the *cytC* and *moeA* genes encode for the cofactor cytochrome C and the molybdenum cofactor biosynthesis protein [30].

Numerous molecular mechanisms of toxicant tolerance have developed in microbes, enabling their growth and metabolism in natural or artificial stress conditions. These, in turn, execute their role in mineralization, redox transformation, complexation, and the decomposition of xenobiotics, metals, metalloids, and organic and inorganic wastes [31], thus acting as ecological restoration agents.

The stressed environments generally bear a combination of toxic agents, which exert their toxicities on the exposed organisms. However, the toxicity incurred on the organisms does not purely depend on the environmental conditions, as the genetic composition plays an essential role in providing tolerance or sensitivity [32]. The ecotoxicological studies regarding single or multi-toxicant-contaminated environments on biota provide important information about the impact and interactive possibilities on the exposed populations. In ecotoxicological bioassays, sensitive or tolerant organisms are used as target organisms to estimate the extent and mechanism of toxicity of a single or multi-toxicant environment. Regarding bacteria as the target organism, the popular ecotoxicological tests include the estimation of toxicity in terms of growth inhibition, reduction in enzymatic processes, bioluminescence, etc. [33,34,35,36,37,38]. Further statistical modeling is employed to understand the kind of interaction between the co-existing toxicants on the test organism and also to provide a predictive relationship between the toxicants [39,40,41].

The two most accepted ecotoxicological concepts are concentration addition (CA) and independent action (IA) [42,43,44]. The CA is relevant if only one toxicity mode applies to all the toxicants in a mixture. However, if different modes of toxicity are employed, IA is applicable. The ease of toxicant load estimation and the simple calculation of the CA model make this model more popular and convincing to the scientific community, in which the toxicity of a mixture is estimated based on individual toxicities of different toxicants [42,45]. Moreover, as the observed toxicities of a mixture are infrequently above the CA expected, it is considered a prudent first tier for environmental risk assessment [46]. In the CA concept, the toxicity units (TUs) of each toxicant are estimated, with the ratio of the toxicant concentration and IC_50_ value, followed by the summation of individual TUs to estimate the expected TUs of the mixture [47]. The observed toxicity values provide information about the additive and subtractive toxicities for synergistic or antagonistic interactions [34].

Several researchers have reported on the individual toxicity of arsenic and fluoride, in terms of reducing or inhibiting growth and reducing catabolic activity in microorganisms [48,49]. However, a lack of data exists regarding the impacts of combined exposure to fluoride and arsenic on microorganisms. In this respect, the study of tolerance to fluoride in combination with arsenic can provide insight into the natural stress impact of toxicants and the subsequent effect on microbial communities.

The ecotoxicological approach of As and F interactive toxicity estimation on bacteria formed the basis of this study, in which the arsenic tolerant *Microbacterium paraoxydans* IR-1 was used as the target organism. The strain IR-1 was previously isolated in our laboratory and was reported as an arsenic (As III) hyper-tolerant bacteria [50].The importance of using bacteria of the genus *Microbacterium* as a target organism is understood by its significant ecological role. The bacteria of the genus *Microbacterium* form an ecologically important entity, with extreme tolerance to environmental contaminants and great potential for the bioremediation of toxicants [50,51,52,53,54,55,56].

The extreme and diverse contaminant tolerance instigated the investigators of this research to study the *Microbacterium paraoxydans* strain IR-1’s tolerance to fluoride, a major groundwater contaminant in the region. Subsequently, the impact of the co-exposure of As and F was estimated by experimental and modeling studies. The toxicity unit model [34] was adopted to determine the toxicity incurred per unit of concentration increase in toxicants, in single and combined exposure groups.

## 2. Materials and Methods

### 2.1. Bacterial Strain

*Microbacterium paraoxydans* strain IR-1, which has been studied for its hyper-tolerance to arsenic (As III), was used in this study. The isolation and characterization of this strain were described in our earlier study [50]. The 16S rDNA sequence has been deposited in GenBank with the accession number KP730604. The strain IR-1 was grown and maintained in nutrient broth (peptic digest of animal tissue 5 g/L, sodium chloride 5 g/L, beef extract 1.5 g/L, yeast extract 1.5 g/L, pH 7.4 ± 0.2) at 37 °C for 24–48 h at 120 rpm in a shaker incubator (Genei).

### 2.2. Estimation of Fluoride Content in Soil

The estimation of the fluoride content of the bacterial isolation source (soil) was performed by following the method given by [57], using an Orion ion analyzer (Orion, Seattle, WA, USA). It consists of a cell with an ion selective electrode and a calomel reference electrode, used to determine the cell potential of standard fluoride solutions. The standard fluoride solutions (MERK MILLIPORE, Burlington, MA, USA; supelco-cat no-119814) of 0.1, 1.0, 10.0, 100, and 1000 ppm were prepared, and the pH was adjusted to 5.35 using a total ionic strength adjusting buffer (TISAB-MERK supelco cat no-89465), followed by the determination of the cell potential of each standard solution. The standard calibration graph was constructed by plotting the cell potential versus log (F), which was used to estimate the unknown fluoride concentration in the soil sample (1 g of soil sample in 50 mL distilled water). The detection limit of the instrument was 0.025–500 ppm.

### 2.3. Dose–Response Relationship Evaluation

To establish the dose–response relationship of toxicants (As and F) on the growth of strain IR-1, it was grown in nutrient broth for 24–72 h and supplemented with increasing doses of the toxicants in three groups, along with a control, to which no toxicant was added (Table 1). The growth in each group was measured in terms of optical density at 600 nm using a double beam spectrophotometer (Schmatzu UV-1800) in a quartz cuvette with 99.5% accuracy, and each group’s minimum inhibitory concentration (MIC) was determined [50]. The toxicants were weighed accurately using a Sartorius weighing balance (Model no: BSA224S-CW) with a detection limit of 0.1 mg.

Determination of Inhibitory Concentration. The toxicant concentration which resulted in a 50% inhibition in growth, i.e., the inhibitory concentration (IC_50_) for each group, was calculated. The inhibition in growth with the supplementation of toxicants (As/F) was calculated as a percentage, with respect to the control (100% growth). The average growth inhibition values at increasing doses were subjected to regression analysis, to draw a linear relationship between the toxicant concentration (As/F) and the percentage of inhibition. Further, the IC_50_values of each group (I, II, and III) were deduced from the regression line. The IC_50_ values of groups III and II were compared statistically using Student’s *t*-test [34,58].

Estimation of Toxicity Units (TUs) of Toxicants. In the present study, the toxicity unit model was used with a modification, namely, keeping one of the toxicants (As) constant at the IC_20_ value (2.5 g/L). In order to compare the toxicity of the two toxicants, it was suitable to express the concentration in terms of the toxicity unit (TU), which was calculated using Equation (1) [34]:

(1) $$\mathrm{TU}=\frac{\mathrm{MIC}}{{\mathrm{IC}}_{50}}$$

To study the interactive effect of the two toxicants (F + As) in Group III, two equations, (2) and (3), were drawn. In these equations, the expected TU (TU_exp_) and observed TU (TU_obs_) were calculated as the sum of the toxicity units of the two toxicants, with respect to the concentration of the toxicant. The TU_exp_ is a measure of the predicted toxicity, calculated by the summation of the individual toxicity of each toxicant at the particular concentration (MIC), whereas the TU_obs_ was calculated by the summation of TU of As at the dose taken and the TU_F+As_ (Group III) at a particular dose, which were experimentally observed (Table 1 and Table 2). In Equations (2) and (3), the toxicity unit of arsenic is shown as TU_As_, fluoride as TU_F_, and TU_F+As_ is the toxicity unit of the combination group (MIC of Group III/IC_50_ Group III):

(2) $${\mathrm{TU}}_{\exp}=\left({\mathrm{TU}}_{\mathrm{As}}\times C_{\mathrm{As}}\right)+\left({\mathrm{TU}}_{F}\times C_{F}\right)$$

(3) $${\mathrm{TU}}_{\mathrm{obs}}=\left({\mathrm{TU}}_{\mathrm{As}}\times C_{\mathrm{As}}\right)+\left({\mathrm{TU}}_{F\;+\mathrm{As}}\times C_{F}\right)$$

Further, the statistical comparison of TU_exp_ and TU_obs_ was performed using Student’s *t*-test with a null hypothesis proposal of no interaction between the toxicants (fluoride and arsenic). The Student’s *t*-test comparison involves the calculation of the difference (TU_diff_) between the expected response and the observed response (Equation (4)), followed by the calculation of the standard error (SE = standard deviation/√N; where N is the number of replicates) and the estimation of SE_diff_ (Equation (5)). Student’s *t*-value was calculated (Equation (6)) and compared at *p* < 0.05–*p* < 0.001, with the degree of freedom calculated using Equation (7) [34,58,59]:

(4) $${\mathrm{TU}}_{\mathrm{diff}}={\mathrm{TU}}_{\exp}-{\mathrm{TU}}_{\mathrm{obs}}$$

(5) $${\mathrm{SE}}_{\mathrm{diff}}=\sqrt{{\left({\mathrm{SE}}_{\exp}\right)}^{2}+{\left({\mathrm{SE}}_{\mathrm{obs}}\right)}^{2}}$$

(6) $$t=\frac{{\mathrm{TU}}_{\mathrm{diff}}}{{\mathrm{SE}}_{\mathrm{diff}}}$$

(7) $$\mathrm{Degree}\;\mathrm{of}\;\mathrm{freedom}\left(d.f.\right)=d.f._{\left(\exp\right)}+d.f._{\left(\mathrm{obs}\right)}$$

### 2.4. Determination of pH

The pH of the medium was also measured in all the groups at the 24 h interval, using a pH meter (Electronic India, Panchkula, India; digital pH meter model-III E) calibrated with a pH 4 and a pH 7 buffer [60,61]. The pH values of Group III were compared statistically with Group II, Group I, and the control group, using Student’s *t*-test [58].

## 3. Results

The isolation source (soil) of the bacterium *M. paraoxydans* strain IR-1 was found to be contaminated with 40.43 mg/kg of fluoride (from the present study) and 84 mg/kg of arsenic, as per our prior study [50].

### 3.1. Dose–Response Relationship

**Figure 1:**
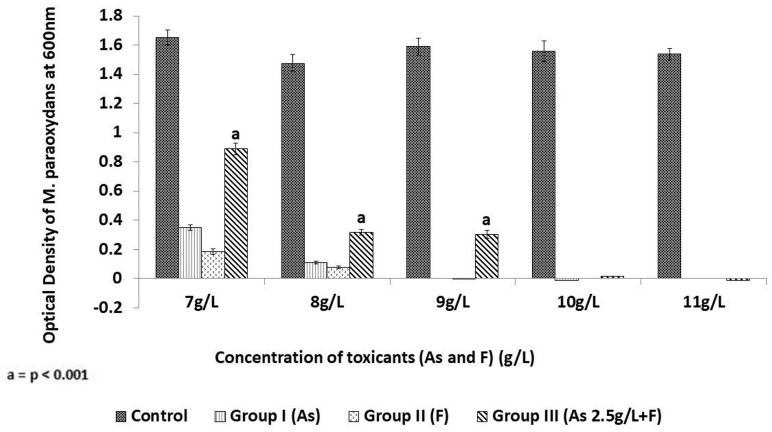
Comparative analysis of fluoride and arsenic toxicity on the growth of *M. paraoxydans* strain IR-1 (in 48 h). The figure depicts the maximum growth in the control group (without any toxicant—100% growth), and a gradual decrease in the growth of the strain when exposed to arsenic (Group I) and fluoride (Group II) individually. But there was a highly significant (*p* < 0.001) increase in the growth of the strain in the combination group (Group III—As + F,) as per the statistical comparison using Student’s *t*-test with Group I and Group II. All the experiments were performed in triplicate and error bars denote standard error.

**Figure 2:**
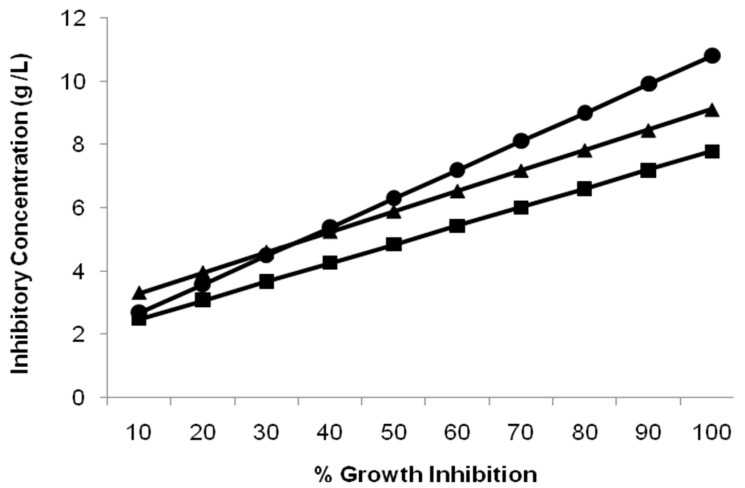
Regression lines representing the growth inhibition of *M. paraoxydans* IR-1 in presence of arsenic (Group I, ■), fluoride (Group II, ▲), and the combination (Group III, ●). The inhibitory concentration of the combination group was higher than the individual exposure to arsenic and fluoride. IC_50_ values for all the three groups were calculated from the respective regression line.

The dose–response relationships of all the studied groups are represented in Figure 1 and Figure 2.

### 3.2. Determination of Inhibitory Concentrations

The dose relationship graph (Figure 1) depicts a toxicant dose-dependent decline in IR-1 growth, with a MIC of 9 g/L for both As (Group I) and F (Group II). Interestingly, in the presence of a constant dose of As (2.5 g/L), the MIC for F was estimated at 11 g/L, as seen in Group III. IC_50_ values for all the three groups were calculated from the respective regression line (Table 2; Figure 2), which also shows a similar pattern, with higher IC_50_ values in Group III (6.322 ± 0.0279 g/L) compared to Group II (5.91 ± 0.01 g/L).

The present study focuses on the combined exposure of two toxicants, i.e., fluoride and arsenic on the growth of the bacterial strain *M. paraoxydans* IR-1. In the combination (F + As) group, the MIC increased by 2 g, compared to fluoride group, along with a highly significant (*p* < 0.001) increase in the IC_50_ values (Table 2 and Figure 2). These results suggest that the presence of arsenic in the medium reduces the toxic impact of increasing doses of fluoride, enabling the strain’s survival even an increased fluoride dose (Figure 1).

### 3.3. Estimation of Toxicity Units of Toxicants

The calculated toxicity units (TUs) of all three groups, as shown in Table 2, was used to estimate the expected and observed toxicity unit (Table 3).The final comparison was undertaken by estimation of TU_diff_ and the *t*-value calculation, which compares the expected toxicity units (TU exp) and observed toxicity units (TU_obs_) of Group II (F) and Group III (F + As). The measurement of toxicity units (TU) provides a fairly good idea of the extent of the toxicity incurred upon the biological agent per unit concentration of toxicants.

Here, the estimation of TU difference of expected toxicity units (TU_exp_) with the observed toxicity units (TU_obs_). in the case of combined exposure to both toxicants (Group III) was found to be highly significant (*p* < 0.001). This shows that the presence of arsenic in the growth medium of bacterium IR-1 can reduce the toxicity levels of fluoride at each successive dose. This significant difference in TU_exp_ and TU_obs_ is considered antagonism, with a reduction in the toxicity of fluoride due to the presence of arsenic.

As per the model of toxicity units used in the present study, if there is no interaction between the toxicants, then the toxicity of the mixture would be determined by the toxicant with the greatest number of TUs present. If the two toxicants have a synergistic interaction, then the toxicity in the combination group would be calculated via the summation of the individual TUs, whereas in an antagonistic interaction, the toxicities of the combination group should be lower than the individual TUs. On the basis of the results obtained in the study, as shown in Table 3, the observed toxicity of the combination group (TU_obs_) was significantly lower than individual expected toxicities (TU_exp_) to the strain IR-1, thus establishing antagonism. Hence, it can be suggested that the presence of arsenic in media reduces the toxicity of fluoride and enables the *M. paraoxydans* IR-1 to survive at high doses of fluoride.

The toxicity of fluoride can be attributed to its chemical nature, as it is the most electronegative of all the elements; thus, it has a strong tendency to acquire a negative charge. Fluoride ions have the same charge and nearly the same radius as hydroxide ions and may replace each other in mineral structures [62]. Fluoride, therefore, can form complexes with a number of cations. Fluoride can act on bacterial cells via its inhibitory action on enzymes, such as glycolytic enzymes, enolase, and heme-based peroxidases. However, the most important factor of fluoride inhibition is its weakly acidic character, as it enhances the permeability of the membrane to protons, thus compromising the function of F-ATPases in exporting protons. This induces cytoplasmic acidification and results in the inhibition of glycolytic enzymes, as reported in a study of oral bacteria [62,63].

The impact of the toxicity of fluoride has been reported in propionate- and butyrate-degrading microorganisms as well as in mesophilic, thermophilic and acetate-utilizing methanogens, which are the main microbial population in wastewater responsible for organic constituent removal; these showed IC_50_ values of fluoride ranging from 18 to 43 mg/L, whereas nitrifying bacteria showed the IC_50_ value of fluoride as 149 mg/L [64]. Other microbial populations, i.e., glucose fermenters, aerobic glucose-degrading heterotrophs, denitrifying bacteria, and H_2_ utilizing methanogens, were able to tolerate a high fluoride concentration (>500 mg/L) [64]. Although fluoride appears to be toxic for microbial growth and metabolism, *M. paraoxydans* IR-1, investigated in this study, is able to resist a comparatively much higher fluoride concentration, and toxic effects appeared only at higher doses, with an IC_50_ value of 5.91 ± 0.01 g/L and a MIC of 9 g/L.

The possible mechanism of fluoride resistance in bacteria has been explored by many researchers. Continuous fluoride stress was found to induce the production of anion-binding ionophores, which can concentrate fluoride and thus reduce its availability [57]. The development of fluoride resistance can also be attributed to genetic change by mutation in the F0-F1 ATPase gene cluster, which has been studied for single nucleotide polymorphism in the fluoride-tolerant bacteria *Streptococcus mutans* [65]. Fluoride stress is known to trigger riboswitches, like the *cbcB* and *eriC* genes, which play a role in inducing the production of anion transporters and other important metabolic pathways [66]. Fluoride resistance in bacteria is also been explained by the evolution of a family of highly selective “Fluc” F-channels that export this inhibitory anion from its cytoplasm [63]. However, the genetic studies were not performed in the present study, but the bacterium IR-1 might apply any of the above-mentioned fluoride tolerance strategies to combat fluoride-induced toxic effects. Various studies on the interactive effects of fluoride and arsenic in higher organisms were summarized in a review [5], wherein the complexity of co-exposure was discussed. In some studies, the synergistic effect of co-exposure was observed, while others reported an antagonistic interaction [67]. The impact of the antagonism of fluoride and arsenic on renal function in a Chinese population was reported [68]. In a brain efficiency study on zebrafish, combined arsenic and fluoride exposure exhibited antagonism in terms of stress markers [69]. However, some research suggests that the dose and duration of arsenic and fluoride exposure also plays a role in determining the synergistic or antagonistic effects [2]. Exposure to As and/or F in a mammalian system has been reported to cause oxidative stress, DNA damage, and perturbations with protein strength [15,70]. Similarly, endoplasmic reticulum stress (ERS)-induced apoptosis has been reported to be the primary mechanism of As- and F-induced injury in H9c2 cells and a rat heart tissue model. Furthermore, the factorial analysis helped to determine the antagonistic toxicological implications in the co-exposure group, with a significant decrease in the expression of the transcription factor CHOP (C/EBP homologous protein), which is involved in ERS-induced apoptosis [65].

The microbial population also shows variability in behavior under toxicant stress. The extent of toxicity incurred on microbes due to toxicant exposure is far more complex, due to the evolution of various tolerance mechanisms. Thus, the exact mechanism of the antagonistic effect of arsenic on fluoride toxicity for the arsenic-resistant bacterium IR-1 is unclear. The antagonism observed in the present study may be because of the formation of AsF_5_, which can reduce the effective concentration of fluoride.

The results of pH estimation of the medium, with and without culture (IR-1), for all the doses, brings us to a possible explanation of this antagonism (Table 4): the growth of *M. paraoxydans* IR-1 and the addition of NaAsO_2_ raises the pH, which can counterbalance the lowering of the pH due to the addition of NaF. Thus, the fluoride tolerance of IR-1 can be attributed to its tendency to raise the pH of the medium, along with its growth, as the acidic character of sodium fluoride is the main factor responsible for its toxicity [63]. Similarly, the rise in pH values with the addition of arsenic to the medium might provide survival benefits to IR-1. Overall, the toxicity of fluoride and its antagonism with arsenic appears to be complex, involving the characteristics of the bacterium *M. paraoxydans* IR-1 and the mineral phases in the medium.

*Microbacterium* are extremophiles with a known tolerance to many metal contaminants, and they have shown potential for use in bioremediation [50,55]. The extreme and varied pollutant tolerance, as well as the diverse habitat survival properties of the genus *Microbacterium*, gives an insight about its possible role in soil recovery and the bioremediation of pollutants in the native soil system. The survival of bacteria under stress conditions can play a crucial role in the mineralization of organic material and the biogeochemical cycling of minerals. However, the precise mechanism of tolerance and the actual role of the strain IR-1 in soil recovery, considering limiting factors and its interactive effect with the native community, need to be explored in soil microcosms in the future.

## 4. Conclusions

The exposure of *Microbacterium paraoxydans* IR-1 to increasing doses of the two toxicants (As and F) undertaken in this study resulted in a gradual decline in growth, with a MIC of 9 g/L for each toxicant. Interestingly, in the combination (F + As) group, the MIC increased by 2 g, compared to the fluoride alone group, along with a highly significant (*p* < 0.001) increase in the IC_50_ values. Further, the toxicity unit model provided the statistical basis for understanding the interactive effect of both toxicants on *M. paraoxydans* strain IR-1. The highly significant (*p* < 0.001) difference (TU_diff_ =1.47) between the expected (TU_exp_ = 21.60) and observed toxicities (TU_obs_ = 20.32) helped to infer that the presence of arsenic in the medium exhibits an antagonistic effect on fluoride toxicity to the bacterium. The significance of the study lies in the unique fluoride tolerance property of *M. paraoxydans* IR-1, which seems to provide support to its comparative growth and preponderance in stressed geological systems. This, in turn, can contribute to the bioremediation and recovery of degraded land systems via the detoxification, removal, and degradation of toxicants.

## Figures and Tables

**Table 1:** Groups used in this study, with varying doses of toxicants (As and F) in nutrient broth inoculated with a pure culture of *M. paraoxydans* strain IR-1.

Groups	Toxicants	Doses of Toxicants Added in Nutrient Broth
Control	No toxicant	Bacterium grown without any toxicant
Group I	Sodium arsenite (NaAsO_2_) (Himedia)	0–9 g/L
Group II	Sodium fluoride (NaF) (Himedia)	0–9 g/L
Group III Combination group	Combination (F + As): ⮚ Sodium fluoride ⮚ Sodium arsenite	0–11 g/L 2.5 g/L (Constant)

**Table 2:** Toxicities of As group, F group, and As + F combined group on *M. paraoxydans* IR-1.

Groups	Toxicant	MIC (g/L)	IC_50_ (g/L)	CV (%)	TU
Control	No toxicant	-	-	-	-
Group I	As (0–9 g/L)	9	4.83 ± 0.025	0.88	1.86 ± 0.01
Group II	F (0–9 g/L)	9	5.91 ± 0.01	0.33	1.52 ± 0.003
Group III	As (2.5 g/L) + F (0–11 g/L)	11	6.32 ± 0.028	0.77	1.42 ± 0.006

As = arsenic; F = fluoride; MIC = minimum inhibitory concentration; CV = coefficient of variance; TU = toxicity unit.

**Table 3:** Statistical analysis of the F and As + F combined groups’ interaction.

TU_exp_	TU_obs_	TU_diff_	S.E._diff_	*t*-Value	Table Value at df = 6	Inference *p* < 0.001
21.60 ± 0.12	20.32 ± 0.09	1.47	0.15	8.71	5.96	Antagonistic

**Table 4:** pH values of media supplemented with fluoride individually or in combination with arsenic and subject to inoculation with a culture of *M. paraoxydans* IR-1.

	Toxicant	Doses of NaF (g/L) Supplemented in Nutrient Broth										
		1	2	3	4	5	6	7	8	9	10	11
pH (without IR-1)	NaF	7.41 ± 0.20 ^b^	7.46 ± 0.24	7.44 ± 0.13 ^b^	7.49 ± 0.2	7.64 ± 0.15 ^b^	7.65 ± 0.14	7.63 ± 0.12	7.97 ± 0.21	7.78 ± 0.05 ^b^	7.88 ± 0.08	7.85 ± 0.12
	NaF + As	8.39 ± 0.23	8.38 ± 0.09	8.37 ± 0.02 ^b^	8.53 ± 0.18	8.67 ± 0.13 ^b^	8.70 ± 0.26	8.88 ± 0.02 ^a^	8.85 ± 0.121	8.96 ± 0.07 ^b^	8.93 ± 0.23	8.90 ± 0.02 ^a^
pH (with IR-1)	NaF	7.87 ± 0.07 ^a,b^	7.79 ± 0.12 ^a^	7.85 ± 0.15 ^a,b^	7.85 ± 0.28 ^a^	7.93 ± 0.08 ^a,b^	7.92 ± 0.17 ^a^	7.91 ± 0.13 ^a^	7.82 ± 0.16 ^a^	7.91 ± 0.04 ^a,b^	7.87 ± 0.02 ^a^	7.92 ± 0.11 ^a^
	NaF + As	8.45 ± 0.17 ^a^	8.70 ± 0.24 ^a^	8.48 ± 0.06 ^a,b^	8.8 ± 0.09 ^a^	9.04 ± 0.12 ^a,b^	9.14 ± 0.12 ^a^	9.15 ± 0.07 ^a^	9.04 ± 0.14 ^a^	9.31 ± 0.18 ^a,b^	9.27 ± 0.17 ^a^	9.17 ± 0.02 ^a^

pH of culture without toxicant: 8.91 ± 0.064, pH of NB: 7.4 ± 0.02, pH of NB with sodium arsenite (As-2.5 g/L): 8.4 ± 0.18. Each value represents mean ± standard error Significance Levels: ^a^ = *p* < 0.001 (highly significant); ^b^ = *p* < 0.05 (statistically significant). Statistical Comparison: 1. NB vs. NB + As—highly significant; 2. NB vs. culture without toxicant—highly Significant; 3. Culture with NaF vs. culture with NaF + As—highly significant; 4. NaF vs. NaF with culture—non-significant; 5. NaF + Ars vs. NaF + As with culture—non-significant.

## Data Availability

The research data of the study has been provided in the manuscript in figures and tables. The raw data can be provided by the corresponding author on demand.
